# Comparison of Microscopic and Endoscopic Transsphenoidal Surgery for Pituitary Adenomas

**DOI:** 10.7759/cureus.80428

**Published:** 2025-03-11

**Authors:** Recep Savik, Yasar Ozturk, Kivanc Yangi, Ismail Bozkurt, Murat Kalayci

**Affiliations:** 1 Neurological Surgery, Bakirkoy Dr. Sadi Konuk Training and Research Hospital, Istanbul, TUR; 2 Neurological Surgery, Yenimahalle Training and Research Hospital, Ankara, TUR; 3 Neurological Surgery, Prof. Dr. Cemil Tascioglu City Hospital, Istanbul, TUR; 4 Neurosurgery, Medical Park Ankara Hospital, Ankara, TUR; 5 Neurological Surgery, Zonguldak Bülent Ecevit Üniversitesi, Zonguldak, TUR

**Keywords:** brain tumor, endonasal endoscopic transsphenoidal surgery, microscopic, nonfunctioning pituitary adenoma, pituitary adenoma, pituitary adenoma management, transsphenoidal resection

## Abstract

Both microscopic and endoscopic transsphenoidal approaches can be utilized to treat pituitary adenomas or pituitary neuroendocrine tumors (PitNETs). While both techniques have shown comparable beneficial outcomes, challenges remain, and definitive long-term data are lacking. A comprehensive study could help optimize surgical strategies for pituitary tumor management, ultimately improving patient outcomes.

This retrospective study compares the outcomes of microscopic and endoscopic transsphenoidal surgeries for treating pituitary tumors. Fifty-two patients (23 men and 29 women) who underwent surgery between January 2012 and December 2018 were included. Patients were classified into two groups based on the surgical approach: 26 underwent microscopic surgery and 26 underwent endoscopic surgery. Preoperative and postoperative MRI scans, tumor size, and hormone levels were analyzed. The primary outcomes evaluated included the extent of tumor resection, complication rates, hormonal remission, and improvements in visual symptoms.

The results showed no statistically significant difference in tumor resection between the two groups, with total radiological remission achieved in 65.4% of patients in both the microscopic and endoscopic groups. The hormonal remission rate was 81.3% in the microscopic group and 86.6% in the endoscopic group. Complications included transient diabetes insipidus in 25% of patients and rhinorrhea in 9.6%, and no surgical mortality was observed. The endoscopic approach offered advantages such as reduced mucosal trauma and improved visualization, while challenges included the need for a bloodless surgical field. Despite these differences, both techniques demonstrated comparable outcomes regarding tumor resection, complication rates, and hormonal remission.

## Introduction

Pituitary adenomas, or pituitary neuroendocrine tumors (PitNETs), are the third most common primary brain tumors after gliomas and meningiomas. They account for 10-15% of all primary brain tumors [1]. According to recent epidemiological data, the prevalence of clinically significant pituitary adenomas is approximately one in 1,000 individuals [2]. Clinically, pathologically, and endocrinologically, pituitary adenomas differ from other intracranial tumors. Although pituitary adenomas are slow-growing benign tumors, some can extend beyond the sella, invading the optic chiasm, cavernous sinus, sphenoid sinus, and adjacent regions [3,4]. In rare cases, adenomas that invade the cavernous sinus may also infiltrate the adjacent bony structures. Currently, there is no reliable parameter to predict which pituitary adenomas will behave aggressively or recur.

Surgical treatment for pituitary adenomas aims to excise the mass lesion without damaging the anterior pituitary tissue, thus avoiding disrupting the hypothalamic-pituitary axis. This includes regulating abnormal hormone profiles, addressing the metabolic effects of the tumor, and alleviating clinical and neurological symptoms caused by the local mass effect. As in all areas of medicine, significant advancements have been made in the surgical treatment of pituitary adenomas over the years. Initially, surgeons used the transcranial approach to treat pituitary adenomas, but due to its high morbidity and mortality rates, less invasive methods were sought.

Austrian neurosurgeon Hermann Schloffer (1868-1937) invented and performed the first transsphenoidal surgery for pituitary adenomas. Later, legendary surgeon Harvey Cushing (1869-1939) adopted the technique and performed his first transsphenoidal surgery in 1909. Between 1909 and 1929, Cushing frequently preferred the transsphenoidal surgical route, contributing to its increasing popularity. However, as he did not find Schloffer’s technique entirely satisfactory, Cushing primarily opted for the sublabial transsphenoidal route, as described by Theodor Kocher (1841-1917) and A. E. Halstead. After 1927, Cushing advocated for the transfrontal approach for pituitary tumors, arguing that it was a more effective surgical method [3].

In the following years, the transsphenoidal approach became the preferred method due to its minimally invasive nature, ease of technique compared to the transcranial approach, lower morbidity and mortality rates, and ability to bypass the arachnoid space. This approach also shortened hospital stays and improved patient recovery and treatment compliance. As the use of transsphenoidal surgery increased, surgeons began to refine the technique, noting that it allowed for wider excisions and had fewer complications.

The endoscope’s role in this technique is particularly notable for its ability to visualize the anatomical boundaries of the pituitary gland by offering lateral views that cannot be seen with the microscope's direct view. In transsphenoidal surgical procedures, the smaller surgical corridor and the broader field of view provided by the endoscope have led some surgeons to favor its use. In recent years, the increasing use of purely endoscopic techniques and the publication of surgical outcomes have spurred comparative studies between these methods and those employing the microscope. Each technique has its advantages and disadvantages.

Today, for tumors with extensive parasellar and suprasellar extensions, the classical transcranial approach or a two-stage approach (first with a transseptal transsphenoidal approach followed by a transcranial approach) is sometimes preferred [5]. This study aims to retrospectively analyze the differences between microscopic and endoscopic transsphenoidal surgery in treating pituitary tumors.

## Materials and methods

Patients

This study included patients diagnosed with pituitary tumors who underwent microscopic or endoscopic surgery by different surgical teams in the same institution between January 2012 and December 2018, with the choice of surgical approach determined based on the experience of the surgical team. This study was reviewed and approved by the Zonguldak Bülent Ecevit University Clinical Research Ethics Committee, adhering to ethical principles (Protocol No: 2019-73-08/05).

Patients with incomplete data or those who underwent transcranial surgery were excluded from the study. Patient files were reviewed, and preoperative and postoperative radiological images and pituitary hormone levels were collected. Patients aged between 19 and 77, regardless of gender, who were diagnosed with pituitary tumors and underwent surgery had their medical histories and physical and neurological examinations evaluated. Patients with pre- and postoperative pituitary magnetic resonance imaging (MRI) scans and pituitary hormone levels were included in the study. Tumor size was measured in three dimensions on preoperative and postoperative MRI images. The outcomes of 26 patients who underwent microscopic surgery and 26 who underwent endoscopic surgery were retrospectively categorized and compared across subgroups. Cases in which the entire visible tumor was removed were classified as gross-total resection (GTR), while cases where the majority of the tumor was removed but a small portion remained were classified as subtotal resection (STR). In contrast, cases where a significant portion of the tumor could not be excised for any reason were classified as partial resection. Additionally, preoperative and postoperative day 1 pituitary hormone levels were analyzed. Cases showing a decrease in hormone levels compared to the preoperative period were noted as remission-positive, while those without a decrease were recorded as remission-negative.

Statistical analysis

Statistical analysis was performed using SPSS version 20.0 (IBM Corp, Armonk, NY), with descriptive statistical methods used to evaluate the data. The chi-square test was used to compare the distribution of patients among the two groups. The data was expressed as mean ± standard deviation (SD). Findings were evaluated at a 95% confidence interval with statistical significance defined as p<0.05.

Surgical techniques

1. Microscopic Technique

Under general anesthesia, patients were placed in the supine position with a pin-head holder. The body was flexed by approximately 20-30 degrees so that the right shoulder was positioned toward the upper right corner of the table. The head was placed at 15 degrees of flexion in the horizontal plane, with the left ear slightly tilted toward the left shoulder. Both nostrils, the nose, the patient's face, and the abdomen (prepared for fat graft harvesting) were cleaned with an antiseptic solution, and the patient was draped in sterile conditions. The surgical procedure was then performed under the surgical microscope (Zeiss OPMI Neuro/NC4, Carl Zeiss AG, Oberkochen, Germany).

Using a dental needle syringe, a submucosal mixture of 5% lidocaine hydrochloride and 1/200,000 epinephrine was infiltrated into the nasal mucosa, posterior septum, and anterior sphenoid wall to facilitate mucosal dissection and minimize bleeding. A Killian retractor was inserted through the right nostril, and a straight incision was made where the cartilage septum meets the bony septum, after which the nasal mucosa was dissected. The cartilage septum was luxated from the bone and retracted laterally to the left. After the Killian retractor was removed, a Hardy retractor was placed to expose the bony septum. The anterior edge of the vomer and the perpendicular plate of the ethmoid bone were resected using a punch to access the sphenoid sinus rostrum. The cartilage and bones were saved for later use during closure. The anterior wall of the sphenoid sinus was opened with a punch, and after entering the sphenoid sinus, the bone was excised with Kerrison rongeurs to expand the field of view. The sphenoid sinus mucosa was resected, and the sellar floor was exposed. The sellar floor was further expanded using punch and Kerrison rongeurs, and a curved incision was made in the dura using a No. 11 scalpel. A biopsy was taken from the tumor, and depending on whether it was a microadenoma or macroadenoma, the tumor was resected accordingly. Absorbable surgical spongostan was placed in the tumor cavity, and the excised bone and cartilage were used for reconstruction. The nasal septum mucosa was closed, and nasal tampons without cannulas were placed in both nostrils. In patients with cerebrospinal fluid (CSF) leakage, a fat graft taken from the abdomen was placed in the operative field. Lumbar external drainage was applied as necessary. The nasal tampons were removed postoperatively after 48 hours.

2. Endoscopic Technique

Under general anesthesia, a fully endoscopic endonasal transsphenoidal surgical approach was performed (Storz Xenon Nova® 175 Model 20131520, Karl Storz GmbH & Co. KG, Tuttlingen, Germany). Preoperative neuroimaging, including paranasal sinus CT scans, was meticulously reviewed to identify the surgical route to the sella and assess for any associated pathologies. Special attention was given to the distance between the internal carotid arteries (ICAs) in the midline, and the absence of intrasellar or suprasellar vascular pathologies was confirmed. Each patient used a nasal decongestant spray the day before surgery. The patient was positioned supine with the right side exposed and placed in a semi-seated position. The head was placed at 15 degrees of flexion, tilted toward the left shoulder, and then fixed in the Mayfield head holder. The face, nasal cavities, and abdomen were cleaned with povidone-iodine solution, and the patient was draped in sterile conditions.

A vasoconstrictor was applied to the nasal mucosa. The procedure was performed using a 4 mm, 18 cm-long, 0-degree rigid endoscope with a binostril approach. Anatomical landmarks, including the inferior turbinate and choanae, were identified first. The middle nasal turbinate was located and gently retracted laterally using a dissector to create the surgical corridor. Mucosal bleeding was controlled with cotton pledgets soaked in epinephrine-containing local anesthetic (jetocaine, lidocaine) to achieve vasoconstriction and hemostasis. The upper turbinate was located posteriorly between the septum and the turbinate, where the natural ostium of the sphenoid sinus was found, usually about 1 cm inferior to the upper turbinate. The nasal mucosa was cauterized, and the mucosa over the bony septum was dissected. This procedure was repeated on the other nostril. After exposing the sphenoid ostia, the anterior wall of the sphenoid sinus was opened. The upper boundary of the osteotomy was typically the horizontal line connecting both ostia. The osteotomy was generally expanded laterally with a Kerrison rongeur or microdrill, with the sphenoid crest in the midline. The mucosa covering the sellar floor was cauterized with an endoscopic cauterizer and dissected with a microdissector. After confirming midline orientation, the sellar floor was opened and expanded using a hook or microdrill, exposing the dura.

To avoid damaging the ICA, micro-Doppler was occasionally used to monitor flow. After cauterizing the dura with an endoscopic bipolar cauterizer, it was opened circularly using a No. 11 or 15 scalpel or microscissors, revealing the adenoma. The lesion was removed with ring curettes and microforceps. If CSF leakage was observed after tumor excision, a fat graft was placed over the sella and secured with fibrin glue. If there was no CSF leak, the surgical site was packed with Spongostan and Fibrillar Surgicel, and the operation was concluded. If the arachnoid membrane was widely opened, a lumbar drain was placed before the patient was awakened. Nasal speculums and endoscope holders were not used during the procedure, and no nasal tampon was applied at the end of the surgery.

## Results

The study included 52 patients: 23 male (n=23, 44.2%) and 29 female (n=29, 55.8%). Gender distribution was not statistically significant difference between the two groups (p=0.402). The mean age was 50.2 years in the microscopic group, and in the endoscopic group, it was 50.6 years. When the distribution of patients’ ages between the two groups was examined, no significant difference was found (p=0.567). The most common symptoms at presentation were headache (n=28, 53.85%), visual disturbances (n=19, 36.54%), abnormal growth of hands and feet (n=17, 32.69%), and central obesity (n=8, 15.38%). As shown in Table 1, most cases were clinically diagnosed as non-functioning adenomas (n=21, 10 males, 11 females, 40.4%), with growth hormone being the most frequently secreted hormone (n=19, 10 males, nine females, 36.5%). When the distribution of clinical diagnoses by gender and groups was examined, no statistically significant difference was found between the two groups (p=0.154, p=0.603, chi-square test, respectively).

**Table 1:** Distribution of clinical diagnoses by gender and groups The percentage values in the table are given approximately by rounding the decimal values.

Diagnosis	Gender		Type of Surgery	
	Male	Female	Endoscopic	Microscopic
ACTH-Secreting Adenoma (n=9, 17.31%)	1 (1.92%)	8 (15.38%)	6 (11.53%)	3 (5.76%)
GH-Secreting Adenoma (n=19, 36.54%)	10 (19.23%)	9 (17.30%)	8 (15.38%)	11 (21.15%)
Non-functioning Adenoma (n=21, 40.38%)	10 (19.23%)	11 (21.15%)	10 (19.23%)	11 (21.15%)
Prolactin-Secreting Adenoma (n=3, 5.77%)	2 (3.84%)	1 (1.92%)	2 (3.84%)	1 (1.92%)
GnRH-Secreting Adenoma (n=0, 0%)	0 (0%)	0 (0%)	0 (0%)	0 (0.0%)
Total (n=52, 100%)	23 (44.23%)	29 (55.77%)	26 (50.0%)	26 (50.0%)

Half of the patients underwent microscopic surgery (n=26, 50%), while the other half had endoscopic surgery (n=26, 50%) (Table 1). According to their size, 41 cases were classified as macroadenomas (10-40 mm) (n=41, 78.8%), eight as microadenomas (<10 mm) (n=8, 15.4%), and three as giant adenomas (>40 mm) (n=3, 5.8%). The distribution of cases based on Hardy-Vezina stages was examined, with stage 3 (n=25, 48.1%) and stage 4 (n=15, 28.8%) being the most common. When the distribution of Hardy-Vezina stages among the groups was examined, no significant difference was found between the groups (p=0.084).

According to the resection results evaluated by postoperative MRI, taken on postoperative day 1, GTR was achieved in 34 cases (n=34, 65.38%), STR in 16 cases (n=16, 30.76%), and partial resection was observed in two cases (n=2, 3.8%). No complications were observed in 46 patients. However, one microscopic case (n=1, 1.9%) had persistent bleeding until 12 hours postoperatively, and rhinorrhea was observed in five cases (n=5, three endoscopic, two microscopic, 9.6% in total). Statistical analysis of complications did not reveal a significant difference based on the surgical technique used (Table 2) (p=0.549). Temporary diabetes insipidus (DI) was seen in 13 patients (n=13, eight endoscopic, five microscopic, 25% in total), while one patient had permanent DI (n=1, endoscopic, 1.9%). 

**Table 2:** Distribution of complications by groups The percentage values in the table are given approximately by rounding the decimal values.

Complication	Surgery					
	Endoscopic		Microscopic		Total	
	Number	%	Number	%	Number	%
Bleeding	0	0.0	1	1.92	1	1.92
Rhinorrhea	3	5.76	2	3.84	5	9.6
No complication	23	44.23	23	44.23	46	88.46
Total	26	50.0	26	50.0	52	100.0

According to the pathology results, 49 patients were diagnosed with pituitary adenoma (n=49, 94.23%). In terms of pathology results of the last three patients, one patient’s diagnosis was basaloid-type squamous cell carcinoma (n=1, 1.9%), while the others were found to be consistent with oncocytoma (n=1, 1.9%) and fibrinoid material (n=1, 1.9%). Similarly, when the distribution of pathology results among the groups was examined, no significant difference was found between the two groups (p=0.364).

There was no statistically significant difference in postoperative hormone remission rates between the two surgical techniques (p=0.071) (Table 3). Evaluation based on the extent of resection showed similar outcomes for both approaches (p=0.082) (Table 4). In the endoscopic group, among the 11 patients who presented with visual disturbance symptoms at admission, 9 showed improved vision in the postoperative period, while 2 experienced no improvement in their symptoms. In the microscopic group, nine patients had visual disturbance symptoms at admission; 8 experienced symptom improvement, whereas 1 showed no improvement in visual disturbances. There was no statistically significant difference between the two groups in terms of visual disturbances (p=0.435). The average follow-up period was 32.2 months. Only 11 patients (n=11, 21.15%) were reoperated due to recurrence, and this group was not subjected to separate statistical analysis.

**Table 3:** Evaluation of hormonal remission rates of the patients and distribution between the groups The percentage values in the table are given approximately by rounding the decimal values.

Hormonal Level	Surgery					
	Endoscopic		Microscopic		Total	
	Number	%	Number	%	Number	%
Remission +	21	40.38	15	28.84	36	69.23
Remission -	5	9.61	11	21.15	16	30.77
Total	26	50.0	26	50.0	52	100.0

**Table 4:** Comparison of resection amount via postoperative MRI The percentage values in the table are given approximately by rounding the decimal values.

Resection Rate	Endoscopic		Microscopic		Total	
	Number	%	Number	%	Number	%
Partial resection	0	0	2	3.84	2	3.84
Subtotal resection	9	17.30	7	13.46	16	30.76
Gross-total resection	17	32.69	17	32.69	34	65.38
Number of cases	26	50.0	26	50.0	52	100.0

## Discussion

Rapid technological advancements have also impacted the transsphenoidal surgical approaches. Developed tools such as neuronavigation, Doppler ultrasound, CT, MRI, and endoscopes have significantly contributed to the diagnosis and resection of pituitary adenomas. The use of the endoscope has expanded the surgical field of vision [6,7]. One of the main advantages of endoscopy is that it allows for faster and less traumatic access to the sphenoid sinus. Additionally, it offers a less invasive approach to the pituitary gland, minimizing damage to surrounding tissue.

Furthermore, intraoperative imaging quality is much improved. The endoscope causes less mucosal bleeding since it minimizes nasal and septal mucosal damage, providing better visualization, increased magnification, and a panoramic view of the sphenoid sinus anatomy [6]. Its wide and close view allows for easier tumor dissection from neurovascular structures. Since a nasal speculum is not used, there is less damage to submucosal tissues, and it provides a broader view and range of movement laterally [8]. Its optical features surpass the microscope's, offering a clearer view of the optic tubercle, carotid tubercle, and opticocarotid recess [9]. It also allows for visualization of the diaphragm sella and cavernous sinus within the sella turcica. If the appropriate instruments are available, the ability to manipulate tools with both hands is a significant advantage for the surgeon. Using both nostrils allows two surgeons to operate with four instruments. In revision surgeries, the same pathway can be used again, and since the previous endoscopic intervention reduces surgical trauma, the second procedure is expedited. Moreover, close-up visualization aids in distinguishing residual tumors from postoperative tissue changes during reoperations [10].

However, there are also challenges and limitations associated with the endoscopic approach. A bloodless surgical field is most notably required, and the surgeon must undergo specific training [11]. Some traditional microsurgical instruments are incompatible with the endoscope. These challenges vary depending on the mobilization capacity of each tool. A frequent issue encountered during endoscopic surgery is lens contamination due to blood or fogging, which can be resolved with endoscopic irrigation [12]. Although endoscopic instruments have limited maneuverability, technological advancements are gradually addressing this problem. Another issue arises with angled endoscope use, especially with 30- and 70-degree endoscopes, where the surgeon may lose orientation, confusing their axis with the viewed axis. Finally, issues with monitor quality may affect the image on the screen. Endoscopy facilitates quicker access to the lesion and enables tumor removal via the same pathway. Regardless of the approach used (middle meatus, superior meatus, or nasal septum), transnasal endoscopy allows for earlier access to the sphenoid sinus. For otolaryngologists, the main advantage of transnasal hypophysectomy is the minimal occurrence of nasal perforation, saddle nose, and dysosmia. Furthermore, using an endoscope allows for direct postoperative observation of the wound area. The packing in the sellar region can be easily visualized, and minor local infections and small CSF leaks can be detected [13].

The endonasal endoscopic surgery differs from microscopic transsphenoidal surgery in several ways. The visual tools used in the operation are different. While the microscope provides a three-dimensional image, the endoscope offers a broader and closer view, although it is not three-dimensional and does not provide depth perception. In pure endoscopic procedures, a nasal speculum is not used, so the approach is endonasal, whereas the traditional approach is transnasal. The classic approach is safe in experienced hands, with a mortality rate below 1.4% [14]. Morbidity remains significant, and complications still occur, primarily due to the size of some lesions and their relationships with adjacent tissues [15].

Ciric et al. categorized surgeons based on their experience and surgical outcomes, dividing them into three groups: those who performed fewer than 200 operations, those between 200 and 500, and those over 500 [14]. Their results demonstrated an inverse correlation between experience and complication rates. The total number of complications is not solely dependent on the extent of tumor resection. Several factors influence the outcome, including the biological behavior of the adenoma, clear visualization of the surgical target, preservation of neighboring anatomical structures (pituitary gland, pituitary stalk, suprasellar cistern, cavernous sinus, and its contents), and the complexity of the anatomical route used to access the tumor. All these factors affect complication rates. The enhanced field of view and clarity provided by the endoscope help reduce complications. Regardless of whether a microscope or endoscope is used, the surgeon's approach is critical in determining the extent of tumor removal and the complication rate [15].

Potential complications associated with the endonasal approach include anesthesia in the upper lip and front teeth, nasal septum collapse, and septal perforation, with rates around 0.3-3%. Inappropriate superior nasal septum dissection can lead to anosmia, maxillary diastasis, and hard palate fractures due to excessive speculum opening. Other complications include orbital fracture, injury to the cribriform plate, subsequent CSF leakage, and bleeding from the mucosal branches of the sphenopalatine artery. Additional complications include mucocele, sphenoid body fracture (which may injure the optic nerve and carotid artery), and sinusitis. The lower rate of complications compared to the microscopic approach is attributed to a smoother opening of the sphenoid sinus without retractors. Potential complications in the sella turcica include subarachnoid hemorrhage, vasospasm, tension pneumocephalus, and CSF leakage. Injuries to the suprasellar and parasellar regions can result in coma, death, vision loss, carotid artery injury, and sixth nerve damage. Endocrine deficiencies of varying degrees may also occur postoperatively, and hormone replacement therapy may be necessary in permanent cases [16-18].

The endoscope has two potential limitations. First, the working channel used to access the sella is different and shorter. The use of a transsphenoidal speculum may offer an advantage. Second, the endoscope may be insufficient in cases of significant bleeding. In such cases, the sphenoid ostium may need to be widened by 1.5-2 cm, allowing the endoscope to maneuver freely and accommodate two instruments alongside it [19,20].

One of the potential benefits of endoscopic surgery is the improved visualization of the surgical field throughout the procedure. Compared to the conical view of the microscope, which is limited due to lens placement and external lighting (restricted by the retractor or nasal passage), endoscopes can be advanced through the nasal route to the sphenoid sinus and sella. By using wide-angle 0-, 30-, 45-, and 70-degree endoscopes, the surgeon can obtain a significantly wider view, even in corners outside the microscope's field. Improved imaging allows for a broader resection and enhanced safety. Additionally, the absence of a sublabial incision and nasal tissue retraction minimizes nasal mucosal trauma and aids in postoperative healing. Consequently, transnasal approaches to the suprasellar cistern and third ventricle facilitate the removal of large macroadenomas [21].

Endoscopic surgery offers minimal damage to the nasal cavity and reduces postoperative morbidity. Recent advancements in endoscopy promise even better imaging and angled endoscopes allow for complete visualization of the nose and paranasal sinuses. The endoscope better distinguishes between normal glandular tissue and tumoral tissue. It is a minimally invasive surgery with fewer complications. Nonetheless, the endoscopic approach also has its shortcomings, the most critical being the need for a bloodless surgical field and specialized training. Endonasal surgery does not require sublabial or nasal incisions. Moreover, there is no need to raise a septal mucoperichondrial flap. This can help prevent orodental, septal, and paranasal complications. Similar findings have been demonstrated in the literature [10,22]. 

The close and wide field of view of the endoscope allows for removing more tumor tissue, reducing residual rates and complications. This is expected to improve success rates in the surgical removal of high-grade macroadenomas with suprasellar extension and cavernous sinus invasion. Remission and complication rates are also linked to the surgeon’s experience and skill. As noted by Ciric et al., true expertise is achieved after 200 operations [14]. In recent years, many surgeons have adopted endoscopic transsphenoidal pituitary surgery, and its outcomes have been compared. A meta-analysis of nine studies using the endoscopic transsphenoidal method (821 patients) between 1993 and 2006 evaluated postoperative outcomes and complications. Seven of the nine studies assessed total tumor removal rates and found an average rate of 78% [21]. Jho's series reported that 77% of 128 patients had macroadenomas, with a total tumor removal rate of 78% [23,24]. In Cappabianca et al.’s series of 146 patients (86% of whom had macroadenomas), the total tumor removal rate was 62%. In comparison, in another study of 300 patients, it was 93%, although macroadenoma data were not provided in the latter [21,22,25].

When analyzing the total tumor resection rates in the 52 patients in our study, total radiological remission was achieved in 17 patients (65.4%) in the endoscopic group. In comparison, residual tumor was detected in nine patients (34.6%). In the microscopic group, insufficient resection was observed in two patients (7.7%), residual tumor was found in seven patients (26.9%), and total radiological remission was achieved in 17 patients (65.4%). The patients with insufficient resection were classified as stage 4, although intraoperatively, total resection was thought to have been achieved. These rates were found to be consistent with the literature [21,25]. However, no statistically significant difference was found between the two groups in our study.

Regarding remission rates in functional adenomas, a meta-analysis of seven studies reported a hormonal remission rate of 79% across all functional adenomas. In Jho’s series of 60 patients with functional adenomas, the remission rate was 70%, while Kabil et al.'s series of 139 patients with functional adenomas reported a remission rate of 87% [23-25]. In our study, the results were evaluated in terms of hormonal remission. Hormonal remission rates were achieved in 13 out of 16 patients (81.3%) in the microscopic group and 13 out of 15 (86.6%) in the endoscopic group. No statistically significant difference was found between the two groups.

Regarding improvement in vision-related symptoms, the literature typically reports improvement rates of over 80%. In our study, 11 patients in the endoscopic group had preoperative vision problems. Full improvement was achieved in six patients (54.5%), partial improvement in three patients (27.3%), and no improvement in two patients (18.2%). In the microscopic group, nine patients had vision problems, with complete improvement observed in six patients (66.7%), partial improvement in two patients (22.2%), and no change in one patient (11.1%). Although better improvement rates were observed compared to the literature, this was not statistically significant between the two groups.

When examining the literature on endoscopic series, rhinorrhea rates were reported as 10% in Jho’s series, 2% in Cappabianca’s series, and 2% in Kabil’s series [21-25]. In our study, the total rate of rhinorrhea was 9.6%, with three cases in the endoscopic group and two in the microscopic group. Regarding transient DI, Jho’s and Cappabianca’s series reported rates of 5% and 4%, respectively, while Kabil’s series reported 4% [22,24,25]. Permanent DI rates were 4% in Jho’s series, 3% in Cappabianca’s, and 1% in Kabil’s. In our study, transient DI was observed in 13 patients (25%), and permanent DI developed in one patient (2%). In the endoscopic group, eight patients developed transient DI, while one patient experienced permanent DI. In contrast, in the microscopic group, five patients had transient DI, with no cases of permanent DI. However, these differences were not statistically significant between the groups. No surgical mortality was observed in our study. In contrast, surgical mortality was reported in one patient each in the Cappabianca and Jho series, while no mortality was reported in Kabil’s series [22-25].

When comparing microscopic and endoscopic transsphenoidal surgical techniques, two critical factors to consider are the availability of endoscopic instruments and the learning curve associated with their use. The cost and accessibility of these instruments must be taken into account, particularly in low- and middle-income countries, where their unavailability may render the technique impractical. Similarly, in rural hospitals lacking endoscopic equipment, microscopic surgery may remain the preferred approach.

Even when endoscopic instruments are accessible, proper training is essential for their effective use, as they involve a steep learning curve. It is generally not recommended to adopt endoscopic techniques without attaining adequate training and expertise. From another perspective, experienced surgeons who have performed pituitary tumor excision using the microscopic approach for many years may face challenges adapting to the newer endoscopic technique. They may even resist this transition and prefer to continue with the microscopic method they are accustomed to.

It is important to emphasize that while minimally invasive surgical techniques are gaining increasing significance and the endoscopic approach offers several advantages, transitioning to endoscopic surgery is neither mandatory nor an absolute necessity. In the hands of an experienced surgeon, the microscopic approach remains a viable and effective option, despite its limitations. Rather than adopting the endoscopic technique without sufficient expertise, it may be more prudent to continue using the well-established microscopic method, which has been refined through years of experience.

Our study has some limitations, such as a relatively small sample size and measuring a limited number of variables. Larger sample sizes are needed to assess statistical significance more accurately, which is crucial for the study's power. Additionally, in future studies comparing these two methods, measuring as many variables as possible is essential to ensure that no factor is overlooked and to allow for a more accurate comparison of the techniques. Another limitation of our study is the recurrence observed in 11 cases (n=11, 21.15%). This is likely due to the relatively high number of cases in which only partial resection or STR was achieved, and the fact that the ultimate goal of GTR could not be attained in every case. On the other hand, given the retrospective nature of our study, evaluating these findings through prospective studies, if possible, would be highly valuable for enhancing the reliability and objectivity of the results.

The long-term effects of endoscopic techniques on tumor control remain an unanswered question, and this will remain the case without sufficient follow-up in a larger series. Currently, there is no large prospective randomized trial comparing endoscopic and traditional microscopic transsphenoidal surgery. The challenges in conducting such a study include difficulties in recruiting a sufficient number of patients to achieve statistical power, issues with randomization, heterogeneity in disease presentation and healthcare centers, and variability in outcome definitions. The non-randomized, observational nature of the current literature is associated with various methodological issues in retrospective studies, such as publication bias, selection bias, insufficient data, and lack of standardization within studies. These challenges highlight the need for prospective studies in the future.

## Conclusions

Endonasal transsphenoidal surgery for pituitary adenomas is safe and effective when performed using either technique. Given that endoscopic techniques are relatively new and not yet widely integrated into neurosurgical training, we believe that endoscopic pituitary surgery should not be performed until surgeons have gained sufficient experience and access to the necessary endoscopic equipment. However, as endoscopic procedures performed by surgeons with adequate experience and proper equipment are associated with lower complication rates and high surgical success, these approaches in neurosurgery are expected to increase. It should be remembered that endoscopes and microscopes are tools that facilitate the surgeon's work. The most critical factor in the success of surgery is the surgeon's clinical experience and surgical skill. Prospective randomized studies with larger patient numbers will significantly contribute to comparing both techniques.
